# Suckling Induces Differential Gut Enzyme Activity and Body Composition Compared to Feeding Milk Replacer in Piglets

**DOI:** 10.3390/ani12223112

**Published:** 2022-11-10

**Authors:** Charlotte Amdi, Marie Louise M. Pedersen, Christina Larsen, Joanna Klaaborg, Andrew R. Williams, Johannes Gulmann Madsen

**Affiliations:** 1 Department of Veterinary and Animal Sciences, Faculty of Health and Medical Sciences, University of Copenhagen, Grønnegårdsvej 2, 1870 Frederiksberg, Denmark; 2 Pig Research Centre, Danish Agriculture and Food Council, Axeltorv 3, 1609 Copenhagen V, Denmark

**Keywords:** artificially reared, conventional reared, body composition, enzyme activity, intestinal health, intestinal morphology, milk replacer

## Abstract

**Simple Summary:**

Large litters have resulted in a surplus of piglets, and there is therefore a need for alternative management tools. Currently, nurse sows (i.e., sows rearing surplus piglets) are the primary solution; however, this means that the sows are contained for a longer period. In addition, a larger proportion of small piglets are born with special needs i.e., extra warmth, energy, etc. This makes it important to investigate alternatives to normal rearing practices in order to ensure piglet welfare to decrease mortality and morbidity in the farrowing unit, as well as in the weaner unit to assure the optimal welfare in pig production. The study showed that piglets could be artificially reared without detrimental effects on the immune system and growth; however, there is a need for further research on optimizing the nutrient composition for artificially reared piglets and the subsequent consequences at weaning.

**Abstract:**

The aim of this study was to investigate differences in growth, hematology, metabolism, small intestine (SI) morphology, and enzyme activity of sow-reared piglets (SOW) compared to artificially reared piglets (MILK) given milk replacers in two different environments. Thirty-six piglets were selected at birth based on their birth weight; eighteen were kept on a commercial farm, another eighteen transferred to an animal research facility for artificial rearing. Differences were observed in enzymatic activity, with a larger amount of sucrase in the SOW compared with MILK group across the SI. SOW piglets also had a body composition with a larger amount of fat, muscle, and bone mass content. Differences in hematology were observed, suggesting environmental influences, biochemistry differences reflective of the diets given, and finally, an increased dry matter (DM) intake in SOW piglets was estimated. No differences were observed in immune function and only small differences in the gut integrity were found between the two groups. It can be concluded that body composition and enzyme activity can be manipulated through dietary intervention and that an increase in DM during lactation is beneficial for gut function. The study warrants further investigation into what this means for the subsequent weaning period.

## 1. Introduction

Due to selection for hyper-prolificacy, modern sows exhibit large litters [1], where there is a surplus of piglets relative to the sow’s own rearing capacity. In addition, these large litters are characterized by higher piglet mortality, greater birth weight variations, and a relatively high number of low birth weight (LBW) piglets within the litter [1,2]. This means that a large percentage of sows are being used as nurse sows [3] and that there are likely a large proportion of piglets that do not achieve a sufficient milk intake to fulfill their full growth potential. In addition, the weaning weight of piglets in Denmark has steadily been decreasing over the past years [4]. Piglets reared from high-yielding sows can gain up to 250 g/day on average from birth to weaning [5], which is substantially below their biological potential when artificially reared having *ad libitum* access to a bovine milk replacer, achieving an average daily gain (ADG) of 400 g/day [6]. With these large litters, there is a risk that the sow milk yield is insufficient in accommodating the biological growth potential of piglets [7]. 

Therefore, to achieve the maximum growth potential and body weight (BW) at weaning, and again at the transition from the weaner to grower-finisher unit, new solutions and alternative management strategies are needed [8]. Artificially rearing in combination with provision of a milk replacer is considered as an alternative strategy, which makes the piglet growth independent from sow milk yield, ensuring that piglet energy and nutritional requirements can potentially be met, and will likely result in decreased morbidity and mortality due to avoidance of crushing from starvation. It is, however, well-known that sow milk differs in nutrient composition [9,10] compared to bovine milk. Milk replacements often contain bovine milk and vegetable-derived ingredients, which contain lower amounts of fat, and a higher concentration of lactose and proteins not found in sow milk. Pieper and colleagues found that proinflammatory cytokine gene expression was higher in piglets fed a milk replacer compared to suckling sow milk, and they also had deeper crypts and lower lactase activity, all factors that might be associated with immunological, physiological, and morphological changes that are strongly related to their health status and inflammation in the gastrointestinal tract (GIT) [8]. In addition to improving the growth rate, milk replacers can be optimized with ingredients that can influence the intestinal development by affecting the morphology and enzyme activity [8] and thus potentially accelerating gut maturation, probably leading to avoidance of a growth check in the early post-weaning period [11]. The maturation of the gut is crucial for the transitional period from suckling to weaning, leading to a smoother adaptation from sow milk to a vegetable-based diet, ultimately ensuring healthier pigs. 

Several studies have suggested that, in addition to age, the main driver for gut maturation is dry matter (DM) intake rather than a specific nutrient composition [12]. In some conventional pig production systems, creep feed is provided to accommodate the piglets to a vegetable-based diet prior to weaning, thereby potentially also increasing the activity of certain disaccharidases. However, the amount of creep feed that is eaten during the suckling period is often negligible. When artificially reared piglets only have access to a milk replacer, which hypothetically can be composed to promote gut maturation or other physiological aspects, there are greater opportunities to prepare the piglet for a smoother transition from suckling to weaning.

Thus, the aim of this study was to investigate two different cohorts of piglets that were the same age but reared in different environments and explore the overall mechanistic effects of artificial rearing with a milk replacer on growth, hematology, metabolism, small intestine morphology, and enzyme activity compared with conventional rearing at the sow. The hypothesis was that differences in DM between the two cohorts could influence gut maturation, development, and growth, with an increase in DM having a positive effect on gut maturation. 

## 2. Materials and Methods

The study was carried out with respect to animal experimentation and with approval from the Danish Animal Experimental inspectorate, license number 2014-15-0201-00418. The artificial rearing and sampling were performed at the experimental facilities at the University of Copenhagen (UCPH) Frederiksberg Campus.

### 2.1. Animals and Experimental Design

Thirty-six piglets from four sows (parity 2–6, Danish Landrace x Danish Yorkshire DanBred, Copenhagen, Denmark) were included in this study. The 36 piglets were mixed females and males (intact) and selected from a commercial farm on the day of birth based on their birth weight (small, medium, or large) and allocated to one of two treatments groups: (a) reared at the sow on sow milk (SOW) or (b) reared artificially and fed a commercial bovine whole milk powder (MILK). All 36 piglets were kept with the dam from days 0 to 3 on the commercial farm. None of the piglets were tail docked, castrated, or given iron injections. After day 3, the 18 piglets from the MILK group were transported to UCPH for further rearing and were also included in another study [13]. The 18 piglets from the SOW group were kept on the farm, and half were transported to UCPH for further sampling at day 24 and the remaining half at day 25. Piglets from both groups were studied from birth to 24 or 25 days of age.

### 2.2. Experimental Diet

From 3 to 24/25 days of age, the MILK group received a milk replacer diet consisting of a mixture of whey protein (DI-9224; Arla Foods A/S, 8260-Viby J, Viby J, Denmark) and bovine milk powder (26% Milk fat, Arla foods A/S, 8260-Viby J, Viby J, Denmark) with 15% dry matter (DM) (see Table 1). The diet was given 8 times a day according to body weight, (BW) and to reduce the risk of leftovers, this was increased to sixteen times over 24 h on day 14. The conventional group was reared at a sow and only received sow milk. The ingredients and chemical composition are shown in Table 1.

### 2.3. Housing and Management Routines

The piglets from the MILK group were transported to the housing facilities at day 3, where they were placed in cages in groups of two to three piglets for the first few hours in order to learn how to drink from the feeding system. They were then assigned to individual cages (for dimensions, cleaning, milk system, etc., see Amdi et al. [13]). The first three days after arrival, all MILK piglets received antibiotic treatment, which included an oral dose with 1 mL of Amoxicillin (50 mg/mL) (Scanvet), 1 mL of Gentocin Vet. (4·35 mg/mL) (Scanvet), and an electrolyte mixture (Revolyte Nutrition, 2–5 mL, Gunnar Kjems). The SOW group on the farm was not given any creep feed but did have access to the sow’s trough. The piglets from the SOW group were reared by two sows until day 24/25, where 18 piglets were selected and were transported to UCPH for sampling at 24/25 days of age (half on each day). They were placed together in a pen with towels, heat, and water until sampling.

### 2.4. Recordings, Blood Sampling, and Analysis

All piglets were weighed after farrowing using a digital weight (UWE, Bjerringbro Vægte, Bjerringbro, Denmark) and ear-marked according to groups. The piglets from the SOW group were weighed two times per week and the day before arrival, where the MILK piglets were weighed once per day. On day 24/25, blood was sampled upon arrival to UCPH/before tissue sampling. Piglets were held in dorsal recumbency for blood sampling, and by jugular vein puncture, 6 mL of blood was collected with a 22-gauge needle in vacutainer tubes containing EDTA for hematology (Advia 2120 Hematology System, Siemens Healthcare Diagnostics, Munich, Germany), serum for biochemistry (Advia 1800 Chemistry System, Siemens Healthcare Diagnostics), and heparin (BD vacutainer, Franklin Lakes, NJ, USA) for lipopolysaccharide (LPS) stimulation to measure the cytokine concentrations as described by Amdi et al. 2020. Briefly, for cytokine production, the peripheral blood mononuclear cells (PBMC) were obtained from heparinized blood samples, washed, counted, and seeded and then stimulated with either 1 μg/mL LPS (Escherichia coli O55:B5; Sigma-Aldrich, St Louis, MO, United States) or mock-treated with PBS as controls. The PBMC were then cultured for 24 h at 37 °C and 5% CO_2_, before the supernatant was harvested and stored at −20 °C. Cytokine concentrations were then measured by ELISA using commercial antibody pairs according to the manufacturer’s instructions (IL-10 and TNFα—Thermo Fisher; IL-6 and IL-1β—R and D Systems).

### 2.5. Dual-Energy X-ray Absorptiometry Scanning and Post-Mortem Examination

Piglets were anesthetized with an intramuscular injection of a Zoletil mix (Zoletil 50; Virbac, Carros, France) containing xylacin (Narcoxyl 20 mg/mL; MSD Animal Health, Rahway, NJ, USA), ketamine (Ketaminol 100 mg/mL; MSD Animal Health, Rahway, NJ, USA), and butorphanol (Torbugesic 10 mg/mL; ScanVet, Fredensborg, Denmark). They were then placed in a Dual-Energy X-ray Absorptiometry (DEXA) scanner in ventral recumbency with the hind legs extended and the forelegs positioned caudally. The whole body composition was analyzed using the small animal mode (Lunar Prodigy Advance; GE Healthcare, Chicago, IL, USA), providing readings of body fat, muscle mass, and bone mass density. The DXA scanner was calibrated using a QC Phantom according to the manufacturer’s specifications. 

### 2.6. Organ Sampling

After euthanasia with intracardial injection (2 to 3 mL pentobarbital, 200 mg/mL), the liver, spleen, kidneys, lungs, heart, adrenal glands, and brain were sampled and weighed on a precision scale (Radwag, Radom, Poland). The stomach was removed and weighed full and empty, similar to the colon. The small intestine (SI) was weighed and the length was measured, and samples were collected from the proximal, medial, and distal parts of the SI. Similarly, one sample was collected from the colon. The samples later used for enzyme activity analysis were collected from the same four places, whereas the samples for the morphology were taken from the three places in the SI and placed in formalin.

### 2.7. Gut Morphology and Histopathology

The tissue samples were prepared for embedding for further histological analysis, as described by Amdi et al. [13]. The morphological and histopathological items analyzed were villous height, crypt depth, enterocyte height, intraepithelial lymphocytes (IELs) infiltration, IELs score, stromal lymphocytes (SL) infiltration score, epithelium score, brush border score, goblet cells/100 enterocytes, and gut-associated lymphoid tissue (GALT). 

### 2.8. Enzyme Analysis

Assays for the mucosal activity of disaccharidases (maltase, sucrase, and lactase) and peptidases (aminopeptidase N, aminopeptidase A, and dipeptidyl peptidase IV) were performed on SI tissue homogenates (homogenized in 1% Triton X-100) using specific substrates, as described previously [14].

### 2.9. Statistical Analysis

All statistical data analyses were performed in R version 1.0.153—© 2009–2017 (R Foundation for Statistical Computing, Vienna, Austria). All data were tested for normality. The effects on all parameters were evaluated by an unpaired student *t*-test using the group (SOW and MILK) as a fixed variable. The interactions were tested and found insignificant. Initially, the birth weight and BW at day 3 were analyzed as response variables to test if they should be included as covariables. This was, however, not the case, because they were insignificant. Results were expressed with the piglet as the experimental unit. Probability levels below 0.05 were considered significant, where <0.10 was considered as a tendency. 

## 3. Results

### 3.1. Nutrient Uptake

To minimize the risk of diarrhea in the MILK group the amount of milk supplied was supplied restrictively according to metabolic body weight. The estimated feed intakes are shown in Table 2. 

### 3.2. Growth Performance and Body Composition

There were no differences between the two groups in birthweight, weight at day 3, and average daily gain (ADG) from birth to day 3. At day 23, the SOW group was heavier than the MILK group (6.5 kg vs. 4.0 kg, *p* < 0.001), which also corresponded with the ADG from day 3 to 23 (0.22 kg/day vs. 0.12 kg/day, *p* < 0.01) (Table 3). The SOW group had higher levels of fat, muscle, and bone mineral content (BMC) than the MILK group (*p* < 0.01) (Table 3).

### 3.3. Organ Weights and Intestine Length

The weight of the SI and the length of the entire SI, as well as the weight of the colon, kidneys, and lungs were greater in the MILK compared with SOW piglets (*p* < 0.05) (Table 4). The liver and spleen were heavier in the SOW compared with the MILK piglets (*p* < 0.05), and there were no differences between the two groups with respect to full and empty stomach weights (Table 4).

### 3.4. Gut Morphology and Histopathology

No differences were found in villous height, crypts, villous-to-crypt ratio (VCR), enterocytes, IELs, IELs scoring, SL scoring, and GALT between the two groups (*p* > 0.05), except for a higher brush border score in the SOW compared with the MILK group (*p* = 0.02).

### 3.5. Enzyme Activity

Sucrase activity was higher across the proximal, medial, and distal SI in the SOW than the MILK group (*p* < 0.05) (Table 5). There were no differences between the two groups with respect to maltase activity in the proximal or medial parts of the SI. The maltase activity was higher in the SOW than the MILK group in the distal part of SI (21.7 vs. 9.3 U/g, *p* < 0.01) (Table 5). No differences were observed in lactase activity in the proximal SI between the groups. Medial SI lactase activity was higher in the SOW group, and there was a tendency for higher lactase activity in the distal SI (*p* = 0.06) in the SOW group compared to the MILK group. The MILK group had higher levels of maltase and lactase in the colon (*p* < 0.01). With respect to the aminopeptidases, Aminopeptidase A (ApA) was increased in piglets from the SOW group across the SI, Aminopeptidase N (ApN) was increased in the proximal part of the SI, and Dipeptidyl Peptidase IV (DPPIV) was increased in the distal and colon compared with the MILK piglets (Table 6). 

### 3.6. Blood LPS Challenge and Hematology

There was no difference between the SOW and MILK piglets with respect to their plasma/serum cytokine concentration (Table 7). The hematology profile of the piglets in the MILK group showed higher levels of hemoglobin, MHCH, thrombocytes, lymphocytes pct. and lymphocytes mia/L (*p* < 0.05) and tendencies for a higher level of hematocrit and MCH compared with the SOW group (*p* < 0.10) (Table 8). However, the levels of neutrophils pct., neutrophil number per (mia/L), and reticulocytes pct. were higher in the SOW group compared with the MILK group (*p* < 0.05) (Table 8). 

### 3.7. Blood Biochemistry

The piglets in the SOW group had a higher level of albumin, total protein, alanin amino transferase, cholesterol, creatinine, inorganic phosphate, and glucose (*p* < 0.05), whereas the MILK piglets had higher levels of basic phosphatase, aspartate amino transferase, blood urea nitrogen, potassium, and triglycerides (*p* < 0.05) (Table 9). There were tendencies for a higher level of calcium for the SOW group compared to the MILK group (2.83 mmol/L vs. 2.71 mmol/L, *p* = 0.07) and a higher level of magnesium for the MILK group compared to the SOW group (1.11 mmol/L vs. 1.20 mmol/L, *p* = 0.07) (Table 9).

## 4. Discussion

A large number of sows are currently giving birth to more piglets than they can rear on their own, increasing the need for alternative or supplementary dietary management strategies. Knowledge about alternative rearing strategies such as artificial rearing, where piglets have been removed from the sow at a very early age, and the impact of these strategies on piglet health and growth is scarce. Thus, the main objective of this study was to investigate the effects of rearing piglets artificially in addition to providing a milk replacer (MILK) on growth, gut health, and function, as well as hematology and serum metabolite profile, compared with conventionally reared piglets suckling the sow (SOW). As the two rearing environments are confounding, this was an explorative study to investigate the sums of different environmental upbringings on the overall piglet physiology and growth and health parameters. The piglets (both SOW and MILK) came from the same herd and were selected on the same day of birth. 

### 4.1. Nutrient Uptake, Growth, Body Composition, and Organ Weight

A key objective in the preweaning period is to accommodate maturation of the gut and immune system without compromising the daily gain and thereby essentially balancing energy and nutrient requirements for both health and growth. When piglets are given *ad libitum* access to cow milk, they can gain as much as up to 400 g/d [6]. The pigs in the current study were restricted in their access to the milk replacer, as based on previous experience from the research facility aiming to reduce the risk of diarrhea. Therefore, a certain reduced growth rate in the MILK piglets was, to some extent, expected as compared with the SOW piglets. 

The fat content of the milk replacer is of great importance, as it is crucial for survival in newborn piglets [15] and therefore essential when aiming at increasing the survival rate and growth of artificially reared piglets only fed on a milk replacer. This is supported by the comparison between MILK and SOW piglets, where the former ingested less than half the amount of fat per day on DM basis. This consequently led to a relatively lower body fat percentage of only 2.4% in MILK piglets compared with a relative body fat percentage of 14.5% in the SOW piglets. In contrast, other studies have shown that supplementing with a milk replacer contributes to an increased weaning weight. In a study by Dunshea et al. [16], feeding with supplemental skim milk increased growth (223 vs. 291 g/day, *p* < 0.001) between days 10 and 20 of age; thus, by weaning, supplemented pigs were 10% (6.13 vs. 6.74 kg, *p* = 0.038) heavier than nonsupplemented pigs [16]. This is also supported by a study where piglets that were split weaned and fed a milk replacer displayed a greater ADG [17]. Although supplementing with a milk replacer can be of great advantage when aiming at increasing the average litter weight and, to some extent, litter weight homogeneity, piglets obviously still benefit greatly from having access to sow milk. This was also apparent in the amount of bone mineral content that was doubled in the SOW piglets compared with the MILK piglets. The relative organ weights were generally heavier in the MILK group, except for the spleen and liver, which were heaviest in the SOW group, the latter playing a vital role in metabolism. 

The biochemistry profile supports the growth performance parameters. The higher levels of alanine amino transferase, a common marker for liver function and development, and creatinine, a common marker for kidney function, in the SOW group suggest a greater level of the overall metabolism compared with the MILK group. In addition, there was more phosphate in sow milk supporting the higher levels of inorganic phosphate in the SOW group. The higher level of blood urea nitrogen in MILK pigs suggests that the milk replacement based on cow milk is not optimal for the piglets, as more amino acids are being deaniminated and therefore excreted in the urine. The higher level of potassium in the MILK group could also suggest that the sodium–potassium pump is not ideal in bovine milk compared to porcine milk. Taken together, the biochemistry results suggest that improvements can still be done on optimizing the milk supplements used in practice. 

Looking beyond the suckling phase, feeding and management obviously play major roles in how pigs perform with respect to the daily gain and feed efficiency in the weaner, grower, and finisher phase, as well as it influences the carcass characteristics when the pig reaches slaughter weight [18]. However, birth weight has also been shown to be a major factor in determining the post-natal growth performance. Although this parameter is naturally and exclusively influenced by the gestation and the level of crowding during gestation, some studies have indicated that there are possibilities to impact the growth performance, muscle protein synthesis, and gut maturation through certain dietary means in the early postnatal period [19,20]. In this respect, it is also in the early phases that the piglets display the greatest fractional protein synthesis, thus representing a crucial period for the piglet to ingest an optimized diet promoting muscle growth and gut development, both beneficial for the later phases. To achieve the greatest effect of an optimized diet, a controlled provision of feed adapted to fit the piglet’s maximum ingestion capacity is essential. This is, however, largely more manageable in a controlled environment such as rescue decks, where piglets are solely fed on a milk replacer and all piglets hypothetically can receive sufficient amounts of milk for a maximized growth. This could perhaps be more beneficial for smaller piglets, but more research is needed to confirm this. However, artificial rearing as described in this paper is not compliant with the current EU legislation, stating that pigs less than 21 days old must not be weaned (EU PiG Innovation Group, Technical Report Precision Production, Ref. Ares (2021) 70893-05/01/202). For this reason, future research should focus on the minimum days of artificial rearing required to substantially reduce piglet mortality in the early postnatal days and the implications on welfare and behavior. 

### 4.2. Maturation of the Digestive Tract

The SOW group had higher enzyme activity levels of sucrose across the SI and also higher levels of maltase and lactase in specific regions compared with piglets in the MILK group. Maturation of the digestive tract is widely accepted as its increased ability to digest and absorb nutrients from vegetable-based diets during the transition from suckling sow milk to being fed a weaner diet [21]. One indicator of SI maturation is its activity level of digestive enzymes—in particular, disaccharidases; maltase, sucrase, and lactase, respectively—involved in the digestion of maltose, sucrose, and lactose to monosaccharides, as well as the aminopeptidases ApA, ApN, and DPPIV involved in the digestion of peptides to amino acids. To adapt to the shift in carbohydrates from lactose to a mainly starch-based diet, the activities of the two former enzymes are expected to increase, whereas lactase activity diminishes because of sow milk withdrawal. As sow milk contains no carbohydrates except for lactose, the SI maturation is likely due to the greater DM intake in the SOW group compared with the MILK, as supported by previous studies showing that the gut integrity was more dependent on diet intake rather than its composition [9,10]. The SOW piglets were reduced down to 12 piglets per litter at the sow from day 3 after birth, ensuring that all SOW piglets had access to a teat and to prevent mortality in the conventionally reared group. This, however, also meant that the SOW group did have access to more milk than piglets in an average-sized litter with 14 piglets in Denmark, and therefore, growth might be greater than expected, creating an even greater difference compared to the MILK piglets. The MILK group had higher levels of maltase and lactase in the colon; however, this was most likely due to microbial activity rather than colon enzyme activity, as the colon tissue was difficult to rinse before sampling, as discussed in Amdi et al. [13]. Similarly, with respect to the aminopeptidases, all three enzymes in all parts of the SI examined at least displayed numerically greater activity in SOW compared with the MILK piglets. Although, most likely, these differences also attributed to the greater DM intake in SOW piglets, it cannot be excluded that the protein composition, being more complex than that of carbohydrates and differing greatly between sow and bovine milk, plays a role in the activity level of the aminopeptidases. Overall, the results of enzyme activity support the hypothesis that a higher DM intake will increase the gut maturation. 

Examining the morphology of the SI revealed almost no differences between the SOW and MILK piglets, which is in contrast to previous studies suggesting the effects of different dietary interventions on gut morphology and function [22,23,24]. Among the most notable are the negative effects on villous and crypts found to be reduced in height and extended in depth, respectively, immediately after weaning. These findings could not be supported by this study, where the groups only differed with respect to the brush border score, suggesting that the MILK group was not experiencing detrimental effects with respect to the physical properties of the gut when being artificial reared on bovine milk. In agreement, Vergauwen et al. [25] found that piglets artificially reared and fed a milk replacer from days 3 to 19 displayed some changes initially in the gut related to villous atrophy when compared with conventionally reared piglets but that these changes had disappeared by weaning [25]. The piglets in the MILK group were individually housed and preweaned on bovine milk, two factors that potentially could stress their biological system and perhaps even reduce their feed intake. However, this was not reflected in the morphology or enzyme activity observed in this study. 

### 4.3. Influence on the Immune System

Development of the immune system is crucial, especially as a preparation for the transition from the suckling to weaning period, where piglets are further exposed to new pathogens due to mixing with individuals from other litters. A little less than half of the hematology parameters measured differed between rearing strategies, and no differences were found with respect to cytokine concentrations between the MILK and SOW piglets. The MILK piglets displayed greater hemoglobin and MCHC concentrations and a greater number of thrombocytes, as well as a greater number and percentage of lymphocytes. On the contrary, the neutrophil number and percentages, in addition to reticulocytes percentages and absolute numbers, were found to be greater in SOW compared with the MILK piglets. Hemoglobin and MCHC concentrations are used as indicators of anemia, which, in piglets, is often regarded as a result of iron deficiency. Neither the MILK nor the SOW group were given iron injections, and their hemoglobin levels were just as low as observed in other studies where iron injections were also not provided [26] and where both groups would classify as anemic [26,27]. The neutrophil levels in the MILK compared with the SOW group are low, suggesting that they have transferred to the tissue, and when carefully interpreted, the respective relationships between lymphocytes and neutrophils could indicate different stages of maturation of the innate and adaptive immune system between SOW and MILK piglets. The increased level of lymphocytes in the MILK group could suggest a switch towards the adaptive immune system in the MILK piglets compared to the SOW piglets. As all the piglets were selected at the same time point and from the same herd, this does indicate environmental differences. In addition, all the MILK piglets were given antibiotics a few days after arrival due to diarrhea, and this could also have influenced the immune system. 

## 5. Conclusions

In conclusion, the enzyme activity suggests that an increased DM intake is important for preparing the SI for weaning. Piglets that were reared solely on a milk replacer (MILK) had a leaner body composition at weaning age compared with piglets suckling sow milk (SOW). In terms of health and growth, artificial rearing had the greatest impact on the latter, as illustrated by the poor daily gain compared with piglets suckling sow milk, whereas, in contrast, the concentration of inflammatory cytokines and number of cells involved in the immune response did not differ greatly between the rearing strategies. This was likely due to the inadequate composition of the milk replacer, which, however, was dosed to balance the growth and gut capacity and prevent diarrhea but also clearly showed that the MILK pigs were more compromised in terms of energy and nutrient metabolism. However, differences in the antibiotic treatments in the two environments could also have influenced the results. Therefore, it is essential to further investigate the nutritional requirements and gut capacity of piglets in the preweaning period regardless of rearing strategy.

## Figures and Tables

**Table 1:** Nutrient composition of the milk replacer (MILK) compared to a standard sow milk composition (SOW) and analyzed chemical composition (as is) in the MILK group.

	Sow Milk (SOW) ^1^	Milk Replacer (MILK)
Whey protein (DI-9224) (g)	-	30
Arla instant whole milk powder (g) 26%	-	120
Energy, kJ/L	4539.6 kJ	2918 kJ
Protein (g/L)	52	56
Lactose (g/L)	54	48
Fat (g/L)	66	31
Minerals (mg/L)		
Sodium (mg)	-	150
Potassium (mg)	-	390
Chloride (mg)	-	15
Calcium (mg)	-	30
Phosphorus (mg)	-	60
Analyzed content		
DM (%)	-	95.9
Crude protein (%)	-	36.5
Crude fat (%)	-	22.4
Ash (%)	-	5.1
Lactose (g/kg feed)	-	30.9
Starch (% DM)	-	<0.5

^1^ Nutrient composition based on the study by Theil et al., 2007 [12].

**Table 2:** Average daily milk and nutrient intake of sow (SOW)-reared piglets based on estimates from Theil et al., 2007 [12], and average daily milk and nutrient supplement of artificially (MILK) reared piglets.

Average Daily Feed Intake ^1^/Provision ^2^	SOW	MILK
Milk, g/d	1096	880
DM, g/d	169	132
Energy, kJ/d	4310.0	2567.8 kJ
CP, g/d per kg DM	50.15	49.28
Fat, g/d per kg DM	63.16	27.28
Lactose, g/d per kg DM	52.18	42.24

^1^ Based on averages of days 10–13 and days 17–20 [12]. ^2^ Based on the average supplementation at days 10, 17, and 24.

**Table 3:** Growth performance a body composition of sow (SOW) and artificial (MILK) reared piglets.

	SOW	MILK	SEM	*p*-Value
Birthweight, kg	1.2	1.1	0.057	0.15
Body weight day 3, kg	1.6	1.4	0.072	0.15
Body weight day 23, kg	6.5	4.0	0.21	<0.01
ADG birth to day 3, kg/day	0.18	0.16	0.015	0.46
ADG day 3 to 23, kg/day	0.22	0.12	0.009	<0.01
Dual-Energy X-ray Absorptiometry				
Fat, g	1146.5	98.6	167	<0.01
Muscle, g	5314.3	3779.1	201	<0.01
Fat, %	14.5	2.4	0.287	<0.01
Bone mineral content, g	110.46	49.23	4.14	<0.01

**Table 4:** Length of small intestine and organ weights of sow (SOW) and artificially (MILK) reared piglets presented as relative organ weights (g/kg) with standard error of the means (SEM).

Organs	SOW	MILK	SEM	*p*-Value
Proximal, g/kg	1.14	1.53	0.05	<0.01
Medial, g/kg	1.07	1.41	0.04	<0.01
Distal, g/kg	13.11	16.03	0.69	<0.01
SI length, cm/kg	22.61	31.16	0.83	<0.01
Colon emp, g/kg	1.09	1.58	0.048	<0.01
Colon full, g/kg	1.58	2.39	0.10	<0.01
Stomach full, g/kg	1.36	1.63	1.60	0.45
Stomach empty, g/kg	0.62	0.67	0.67	0.18
Kidneys, g/kg	0.69	0.80	0.02	<0.01
Liver, g/kg	2.74	1.39	0.08	<0.01
Lungs, g/kg	1.39	1.71	0.09	<0.01
Spleen, g/kg	0.49	0.40	0.02	<0.01
Heart, g/kg	0.76	0.69	0.03	0.06

**Table 5:** Activity of sucrase, maltase, and lactase in the proximal, medial, and distal parts of the small intestine and in the colon of sow (SOW) and artificially (MILK) reared piglets.

	SOW	MILK	SEM	*p*-Value
Proximal SI, U/g				
Sucrase	9.9	7.5	0.59	<0.01
Maltase	36.1	37.6	3.34	0.73
Lactase	32.4	33.8	2.52	0.69
Medial SI, U/g				
Sucrase	13.0	6.5	0.79	<0.01
Maltase	43.0	47.8	3.82	0.37
Lactase	54.2	28.4	3.61	<0.01
Distal SI, U/g				
Sucrase	4.1	2.1	0.49	0.01
Maltase	21.7	9.3	3.07	<0.01
Lactase	18.4	11.4	2.59	0.06
Colon, U/g				
Sucrase	0.9	2.1	0.44	0.08
Maltase	3.3	17.7	2.71	<0.01
Lactase	3.5	41.8	8.22	<0.01

**Table 6:** Activity of aminopeptidases ^1^ in the proximal, medial, and distal part of the small intestine and in the colon of sow (SOW) and artificially (MILK) reared piglets.

	SOW	MILK	SEM	*p*-Value
Proximal SI, U/g				
ApA	3.77	2.92	0.181	<0.01
ApN	6.11	4.91	0.383	<0.05
DPPIV	2.15	2.13	0.0912	0.85
Medial SI, U/g				
ApA	4.73	3.80	0.25	<0.05
ApN	8.19	7.10	0.475	0.10
DPPIV	2.92	2.65	0.135	0.17
Distal SI, U/g				
ApA	5.23	2.40	0.76	<0.05
ApN	7.97	5.72	1.12	0.17
DPPIV	5.68	2.89	0.828	<0.05
Colon, U/g				
ApA	0.62	5.32	1.49	<0.05
ApN	0.74	1.20	0.24	0.18
DPPIV	0.78	2.92	0.713	<0.05

^1^ Aminopeptidase A (ApA), Aminopeptidase N (ApN), and Dipeptidyl Peptidase IV (DPPIV).

**Table 7:** Blood serum/plasma cytokine concentration in sow (SOW) and artificially (MILK) reared piglets.

	SOW	MILK	SEM	*p*-Value
IL-6, pg/mL	1121.1	742.6	223	0.22
IL-10, pg/mL	91.5	79.7	21.3	0.65
TNFα, pg/mL	1654.5	1076.6	574	0.49
IL-1β, ng/mL	3.4	5.8	2.3	0.40

**Table 8:** The hematology profile of sow (SOW) and artificially (MILK) reared piglets.

	SOW	MILK	SEM	*p*-Value
Total leukocytes, mia/L	6.62	6.62	0.39	0.99
Total erythrocytes, bill/L	3.93	4.33	0.215	0.21
Hemoglobin (HGM), mmol/L	2.72	3.12	0.13	0.03
Hematocrit (HCT), L/L	0.16	0.18	0.008	0.09
MCH ^1^, fmol	0.68	0.73	0.02	0.07
MCHC ^1^, mmol/L	16.27	17.09	0.23	0.01
Thrombocytes	1087.5	1380.9	103	0.05
Mean platelet volume (MPV), fL	14.34	15.82	1.16	0.37
Mean cell volume (MCV), fL	41.86	42.59	0.69	0.45
Mean platelet count, (MPC), g/L	255.82	258.82	2.82	0.46
Neutrophils, pct	30.07	20.46	2.03	<0.01
Lymphocytes, pct	56.62	73.48	2.41	<0.01
Monocytes, pct	2.10	2.51	0.244	0.24
Eosinophils, pct	1.09	2.28	0.73	0.25
Basophils, pct	0.26	0.29	0.05	0.73
Large unstained cells (LUC), pct	0.89	0.94	0.163	0.72
Neutrophils, mia/L	2.60	1.38	0.19	<0.01
Lymphocytes, mia/L	3.74	4.85	0.3	0.01
Monocytes, mia/L	0.14	0.17	0.02	0.28
Eosinophil, mia/L	0.07	0.14	0.04	0.27
Basophils, mia/L	0.02	0.02	0.004	1.00
Large unstained cells (LUC), mia/L	0.05	0.06	0.01	0.55
Reticulocytes, pct (estim)	12.1	4.19	0.76	<0.01
Absolut reticulocyte, mia/L (estim)	449.51	180.14	28.3	<0.01

^1^ MCH = mean corpuscular hemoglobin, MCHC = mean corpuscular hemoglobin concentration.

**Table 9:** Blood serum metabolites of sow (SOW) and artificially (MILK) reared piglets.

	SOW	MILK	SEM	*p*-Value
Albumin g/L	38.80	31.08	0.83	<0.01
Total protein, g/L	54.70	45.85	0.98	<0.01
Basic phosphatase, U/L	1011.89	1641.89	136	<0.01
Alanin amino transferase U/L	32.17	21.55	3.03	0.02
Cholesterol, mmol/L	4.33	2.61	0.74	<0.01
Creatinine, umol/L	80.44	69.06	0.21	<0.01
Iron umol/L	3.68	2.48	0.00	0.12
Inorganic phosphate, mmol/L	2.96	2.2	0.54	<0.01
Aspartate amino transferase U/L	46.17	57.83	0.09	0.61
Blood urea nitrogen, mmol/L	3.55	6.74	16.1	<0.01
Gamma-glutamyl transferase, U/L	26.83	26.38	0.30	0.89
Calcium, mmol/L	2.83	2.71	2.27	0.07
Magnesium, mmol/L	1.11	1.20	0.05	0.07
Sodium, mmol/L	140.55	143.33	0.03	0.15
Potassium mmol/L	4.70	6.16	1.33	<0.01
Glucose, mmol/L	8.17	6.49	0.29	<0.01
Triglyceride, mmol/L	0.80	1.34	0.25	<0.01
Creatine Kinase U/L	1042.5	1406.1	0.08	0.52

## Data Availability

Available on request.
